# Insight into the effects of different oxygen heteroatoms on nicotine adsorption from cigarette mainstream smoke

**DOI:** 10.1038/s41598-023-42188-w

**Published:** 2023-09-15

**Authors:** Phongphot Sakulaue, Kulpavee Jitapunkul, Parinya Inthasuwan, Hiromu Takano, Takafumi Ishii, Kanokwan Kongpatpanich, Kajornsak Faungnawakij, Metta Chareonpanich, Khanin Nueangnoraj

**Affiliations:** 1 School of Bio-Chemical Engineering and Technology, Sirindhorn International Institute of Technology, Thammasat University, Pathum Thani, 12120 Thailand; 2 Sustainable Energy and Resources Engineering, Faculty of Engineering, Kasetsart University, Bangkok, 10900 Thailand; 3 International Research and Education Center for Element Science, Faculty of Science and Technology, Gunma University, 1–5–1 Tenjincho, Kiryu, Gunma 376-8515 Japan; 4 Department of Materials Science and Engineering, School of Molecular Science and Engineering, Vidyasirimedhi Institute of Science and Technology, Rayong, 21210 Thailand; 5 National Nanotechnology Center, National Science and Technology Development Agency, Pathum Thani, 12120 Thailand; 6 Department of Chemical Engineering, Faculty of Engineering, Kasetsart University, Bangkok, 10900 Thailand

**Keywords:** Engineering, Materials science, Chemistry, Environmental chemistry, Materials chemistry, Surface chemistry

## Abstract

Cigarette smoke contains many chemicals, including nicotine, which is harmful and can cause health problems such as carcinogenesis disease, cardiovascular, respiratory, renal, and reproductive systems. Removal of nicotine from mainstream smoke can be done through adsorption with filters or solid adsorbents. In this study, we explored the use of activated carbons for the removal of nicotine from cigarette mainstream smoke. Activated carbons were prepared from dried hemp (*Cannabis sativa*) stem at an activation temperature of 350–550 °C using phosphoric acid as an activating agent. The results showed that the activated carbons with variable surface functional groups and porosity exhibited high efficiency for nicotine adsorption, removing 68–88% of nicotine from cigarette mainstream smoke. Through X-ray photoelectron spectroscopy and temperature-programmed desorption analyses, we identified that oxygen-containing functional groups, particularly carboxylic groups, exhibited a superior ability to adsorb nicotine. The computational analysis with DFT simulations further supported the importance of oxygen-containing surface functional groups in facilitating nicotine adsorption, with the carboxylic group providing the lowest adsorption energy among other functional groups.

## Introduction

Cigarettes are one of the most addictive and relaxing means of people. However, cigarettes also produce smoke, particulate matter, and some chemical toxicants that affect organisms and the environment^1–3^. Smokers can directly inhale the smoke from a cigarette butt, which is called first-hand smoke, and they exhale the smoke that combines with the sidestream smoke from the burning cigarette butt, which is called second-hand smoke^4^. Both types of smoke are produced when tobacco leaves are burned and causes health problems such as respiratory, circulatory, immune system of the body, and long-term effects such as cancer and some lung diseases.

The composition of mainstream smoke is mainly nicotine, carbon dioxide, and polycyclic aromatic hydrocarbons (PAHs)^5^. Nicotine can lead to an increase in blood pressure, heart rate, and blood flow to the heart, as well as an increased risk of stroke^6^. In general, can exist in unprotonated, monoprotonated and diprotonated forms. The non-volatile, protonated forms of nicotine reside only in the particle phase, whereas the volatile unprotonated form is present in both the particle (liquid) and gas phase, and essentially this latter form of nicotine determines the rate and extent of uptake of nicotine during smoking^7–9^. A cigarette butt can block out some of the mainstream smoke. In addition, the addition of additives to the cigarette butt can lead to further adsorption of toxicants. Removal of tar and nicotine from mainstream smoke using zeolite, activated carbon, and oxidized carbon nanotubes as additives was reported^10^. It was found that the oxidized carbon nanotubes exhibited exceptional removal efficiency despite their low specific surface area. Although their adsorption mechanism was unclear, capillary condensation of some ingredients from the mainstream smoke was observed in the inner hole of the oxidized carbon nanotubes and was suggested to be the main reason for their excellent removal efficiency.

Although there are several studies on the adsorption of nicotine on various solid adsorbents^11–23^, most of the works focus on the removal of nicotine in liquid media. There are only a few studies on the adsorption of nicotine in the gas phase^13,15–17,21–23^, which is suitable for the removal of nicotine from cigarette mainstream smoke. Zhou et al*.* reported the removal of toxicants in mainstream cigarettes using a mixture of carbon nanotubes with mesoporous and microporous nanostructures obtained from catalytic pyrolysis of polypropylene and modified montmorillonite nanocomposites^13^. However, the yield of nicotine removal is still lower than the removal of other harmful compounds in mainstream smoke. Although there have been research works reporting the high nicotine removal capacity by using molecularly imprinted polymer^15^, oxidized mesoporous spherical carbon^16^, multiwalled carbon nanotubes (MWCNTs) based thin flexible membrane^17^, sepiolite granules^21^, polydopamine-decorated filter tip^22^, and nanocellulose-SiO_2_ hybrid aerogels^23^, the key factors affecting effective nicotine adsorption are still unclear. The adsorption capacity depends not only on physical adsorption but also on chemical adsorption. In general, physical adsorption ability is mainly due to the surface area and pore volume of the adsorbent, while the surface chemistry of the adsorbent plays an important role in chemical adsorption. Yang et al*.* reported that the adsorption of nicotine in a solution to carbons mainly depends on the surface chemistry of the carbons^12^. They found that phenolic groups could increase nicotine adsorption capacity, while carboxylic groups could hinder the adsorption because they could combine with water molecules though hydrogen bonds and block the pores. Adsorption in the gas phase could overcome this problem, and the carboxylic groups could dominate the adsorption of nicotine. These strategies were also supported by Pi et al*.*^14^, who reported that the increased adsorption capacity of nicotine was due to the higher concentration of acidic surface functional groups of activated carbon. However, to our knowledge, the effect of the type of functional groups on the surface of activated carbon on nicotine adsorption has never been extensively studied.

This study focuses on improving the technique to demonstrate surface-dependent removal of nicotine with activated carbon. In this case, the activated carbons were prepared using hemp (*Cannabis sativa*) stem activated with phosphoric acid (H_3_PO_4_), which enriched the oxygen-containing surface functionalities of the activated carbons. The elemental composition, porous texture, and surface functional groups of the prepared activated carbons were experimentally investigated by elemental analysis, N_2_-sorption measurements, and temperature-programmed desorption (TPD). In addition, the adsorption energy of nicotine molecules with surface-functional groups was calculated based computational analysis using DFT simulations. Here we report the surface-dependent adsorption of nicotine, where carboxylic groups showed the greatest influence on nicotine removal among the other oxygen-containing functional groups. The key contribution of this study is the identification of surface-dependent adsorption of nicotine, with a particular focus on the influence of different oxygen-containing functional groups. The results indicate that carboxylic groups exhibit the highest impact on nicotine removal among the various functional groups investigated. This finding has implications for the design and optimization of activated carbon materials for the efficient removal of nicotine and potentially other similar compounds.

## Experimental

### Preparation of activated carbons

The hemp stem was ground into powder and dried before use. It was impregnated with phosphoric acid (H_3_PO_4_, 85%w/w) at a weight ratio of 1:1 and heated at 120 °C. The impregnated hemp stem was carbonized in a horizontal tubular furnace under a nitrogen flow (200 cm^3^ min^−1^). The activation temperature was between 350 and 550 ℃. The heating rate and holding time were 10 ℃ min^−1^ and 2 h, respectively. Finally, it was washed with RO water and dried overnight at 105 ℃. The as-prepared ACs were labeled as AC(*T*), where *T* is the activation temperature. The use of plant parts in the study complies with international, national, and/or institutional guidelines.

### Characterizations of activated carbons

The amounts of carbon, hydrogen, and nitrogen of the hemp stem and the obtained activated carbons (ACs) were characterized by CHN analysis (CHNS/O analyzer 628 series, Leco Corporation, USA). The porous textures of the samples were determined by N_2_ sorption measurements at − 196 °C (BELsorp MiniX). The specific surface area was calculated using the Brunauer–Emmett–Teller method (*S*_BET_). Total pore volume was estimated from the N_2_ adsorption amount at a relative pressure of 0.99 (*V*_total_). The micropore volume was calculated using the Dubinin–Radushkevich (DR) equation (*V*_micro_)^24^. The obtained ACs were also characterized by Fourier transform infrared spectroscopy (FTIR, Nicole iS50, Thermo Fisher Scientific) in transmission mode (2.5 wt% in KBr). The number of scans and resolution were 16 and 4, respectively. X-ray photoelectron spectroscopy (XPS) measurements were performed using a Kratos AXIS NOVA instrument (Shimadzu Co.). The charge-up shift correction was conducted by setting the C 1s binding energy level of the samples to 284.5 eV. The ACs were also subjected to transmission electron microscopy (TEM, JEOL: JEM 3100F) to investigate pore structure and morphology of the samples.

### Nicotine removal ability test

The ability to remove nicotine was investigated using a laboratory-scale fixed-bed adsorption apparatus (Figure S1). The obtained ACs (20 mg) were filled into the cavity (approximately 1 mm) between two cigarette filters (a 10–mm cellulose acetate filter). Five cigarettes (Red SMS®, Tobacco Authority of Thailand) were lit and then smoked with a pump. The mainstream smoke would pass through the cigarette filter and a Cambridge filter pad (CFP), in which 99% of particles larger than 100 nm were captured^25^. Small particles would pass through the CFP pad and were eventually captured in a methanol solution^26^. Cigarettes were smoked until the ignition reached a length of 50 mm. After that, the CFP was soaked in methanol solution for 30 min and the solution was finally analyzed with a gas chromatography – flame ionization detector (GC–FID) (see Figure S2 and Table S1)*.*

Nicotine reduction ($\%{R}_{\mathrm{nicotine}}$) was calculated using the following equation:

1 $$\%{R}_{\mathrm{nicotine}}=\frac{{C}_{\mathrm{nicotine },\mathrm{ blank}} - {C}_{\mathrm{nicotine},\mathrm{ ACs}}}{{C}_{\mathrm{nicotine },\mathrm{ blank}}}\times 100$$

where ${C}_{\mathrm{nicotine},\mathrm{ blank}}$ is the nicotine concentration of a blank cigarette without ACs and ${C}_{\mathrm{nicotine},\mathrm{ ACs}}$ is that filled with ACs. Normalized nicotine reduction was also estimated by dividing *%R*_nicotine_ by *S*_BET_. Note that nicotine reduction ($\%{R}_{\mathrm{nicotine}}$) was measured in three different batches, with each batch measured three times by GC-FID to ensure reliability of the results.

### Quantitative analysis of surface functional groups of ACs

Temperature-programmed desorption mass spectrometry (TPD-MS) was used to quantitatively investigate the oxygen-containing functional groups of the samples. A detailed description of the experimental setup and procedure can be found elsewhere^27,28^. Briefly, the sample was placed in a graphite holder in a quartz chamber and evacuated to a high vacuum. Once the pressure was below 2 × 10^−5^ Pa, the sample was heated from room temperature to 1600 °C at a heating rate of 10 °C min^−1^ in high vacuum using a high-frequency induction coil. The desorbed gases (CO and CO_2_) were quantitatively measured using a quadrupole mass spectrometer.

### Computational study of oxygen-containing surface functional groups on nicotine adsorption

**Figure 1:**
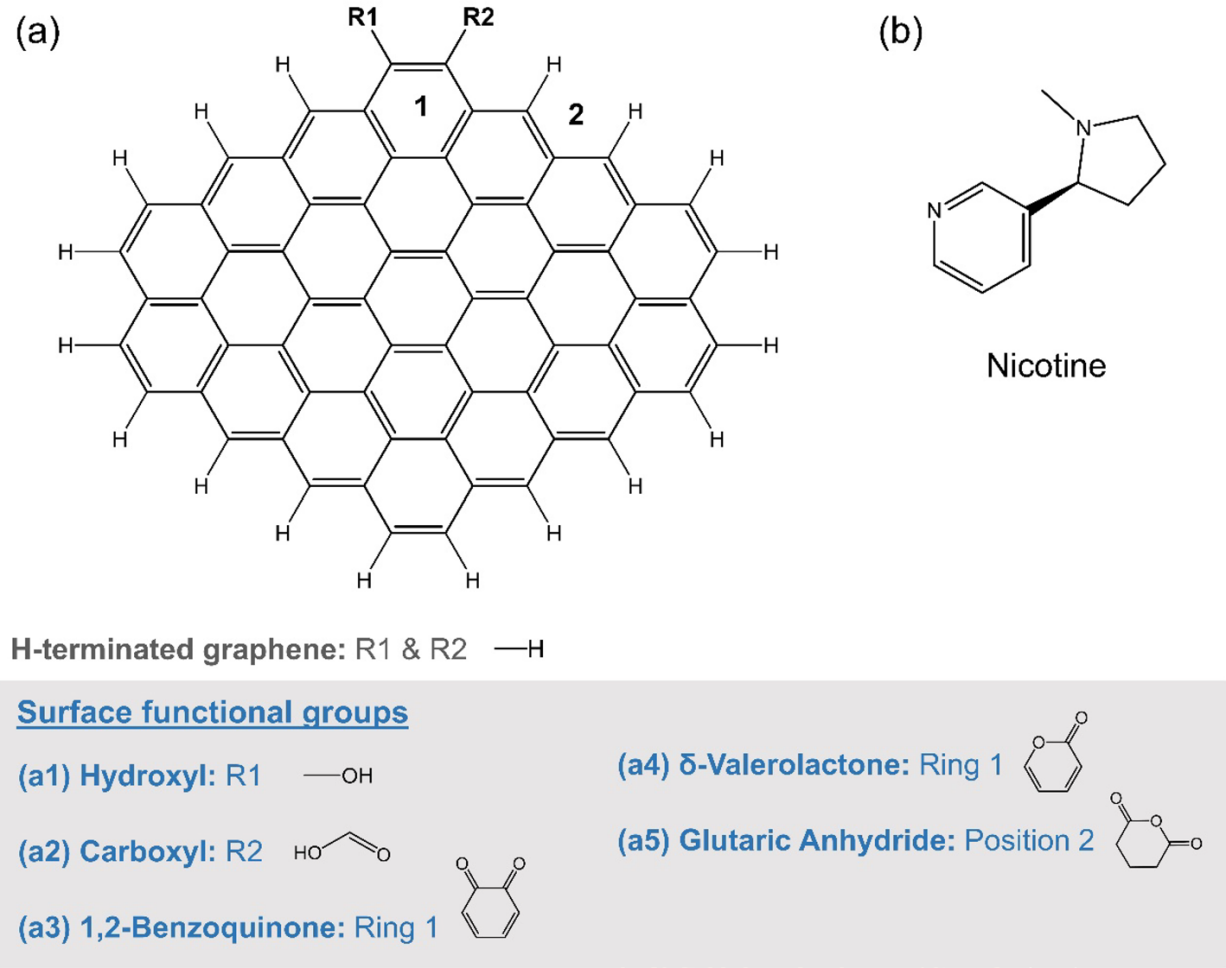
(**a**) Schematic representation of hydrogen-terminated C64 graphene and positions (R1-R2, Ring 1, and position 2) for substitution of oxygen functional groups (a1–a5). (**b**) Chemical structure of nicotine.

The study of surface functional groups on nicotine adsorption was also performed using a computational approach. The hydrogen terminated C64 graphene and the modified C64 graphene with oxygen-containing functional groups (Fig. 1a) were prepared in GaussView 6.0^29^. The structure of the nicotine molecule (Fig. 1b) was downloaded from PubChem with CID 89594^30^. The structures of the graphene-nicotine complex were constructed in the Discovery Studio Visualizer 4.0 program^31^ by placing the nicotine molecule near the edge of the graphene sheet, approximately 3–4 Å away from the terminated hydrogen atoms.

Geometry optimization of all structures was performed in the Gaussian 16 program^32^ using M06-2X functional with 6-31G(d,p) as the basis set to obtain the lowest energy structures. We hereby collect the global energy minimum for the calculation of the adsorption energy (Δ*E*), as shown in Eq. (2).

2 $$\Delta E={E}_{complex}-({E}_{graphene}+{E}_{nicotine})$$

The efficiency of nicotine adsorption between different surface functional groups of graphene was determined based on adsorption energy values and intermolecular interactions.

## Results and discussion

### Elemental analysis and pore characteristic of ACs

The elemental composition of the precursor, and the obtained ACs, are listed in Table 1 along with their porous properties. The hemp stem has a relatively high carbon content, being mainly composed of a woody core containing cellulose, hemicellulose, and lignin^33,34^. Upon carbonization and activation, the carbon content, as well as *S*_BET_ and the pore volumes increase significantly, which is generally observed during chemical activation.

**Table 1:** Carbon, hydrogen, nitrogen content (excluding ash) and porous properties of the precursor and the as-prepared ACs.

Sample	Elemental composition			*S*_BET_^a^ (m^2^ g^−1^)	*V*_total_^b^ (cm^3^ g^−1^)	*V*_micro_^c^ (cm^3^ g^−1^)	*V*_meso_^d^ (cm^3^ g^−1^)
	C (%w w^−1^)	H (%w w^−1^)	N (%w w^−1^)				
Dried hemp stem	46.2	6.0	11.2	–	–	–	–
AC(350)	58.0	2.9	5.0	945	0.47	0.37	0.11
AC(400)	62.3	3.1	6.4	1,660	1.00	0.59	0.41
AC(450)	62.1	2.7	7.6	1,720	1.19	0.57	0.62
AC(500)	63.8	2.0	11.7	1,960	1.48	0.65	0.83
AC(550)	58.3	2.0	11.7	1,880	1.39	0.61	0.78

^a^ *S*_BET_—specific surface area, determined at 0.01 < *P/P*_0_ < 0.05 by the BET method. ^b^ *V*_total_—total pore volume, determined at a *P/P*_0_ of 0.99. ^c^ *V*_micro_—micropore volume, determined by the DR method. ^d^ *V*_meso_—mesopore volume, determined by subtracting the micropore from the total pore volume.

N_2_-sorption measurement was employed to investigate the porous textures of each activated carbon. The *S*_BET_ can be estimated using the BET equation, while the total pore volume is determined at a relative pressure of 0.99, as listed in Table 1. The results show that with increasing activation temperature, both surface area and total pore volume increase up to a certain point (up to 1960 m^2^ g^−1^ at 500 °C) and then decrease slightly. The obtained ACs have a high specific surface area (945–1960 m^2^ g^−1^) and their N_2_-sorption isotherms (Figure S3a) can be classified as a combination between types I and IV, which can attribute to the presence of micro and mesoporosity. This is also consistent with the pore size distribution of the obtained activated carbon shown in Figure S3b, which is dominated by micropores of pore diameter about 1 nm. However, it can be seen that increasing the activation temperature increases the mesoporosity, which is in good agreement with the increase in mesopore volume shown in Table 1.

### Nicotine adsorption ability of ACs

The ability to adsorb nicotine from cigarette mainstream smoke by using the as-prepared ACs was investigated using a laboratory-scale smoking apparatus. The percentage of nicotine reduction ($\%{R}_{\mathrm{nicotine}}$) was calculated as listed in Table 2, where 68 – 88% of nicotine can be adsorbed and the adsorption capacity increases with activation temperature. Since the adsorption capacities of nicotine on the obtained ACs are not only related to the surface area and pore size, but also the surface chemistry, such as surface functional groups, played an important role in chemisorption. As we observed no significant differences in pore structure and morphology of the ACs upon TEM observations (see Supporting Information, Figure S5), we thus normalized nicotine reduction by dividing $\%{R}_{\mathrm{nicotine}}$ by its *S*_BET_ to eliminate the influence of specific surface area on nicotine adsorption. As can be seen in Table 2, the normalized $\%{R}_{\mathrm{nicotine}}$ shows contradict results, suggesting that the adsorption capacity is highly dependent on the surface functional groups.

**Table 2:** Nicotine reduction using the obtained ACs^a^.

Sample	Nicotine reduction ($\%{R}_{\mathrm{nicotine}}$)	Normalized nicotine reduction ($\%{R}_{\mathrm{nicotine},\mathrm{norm}}$)^a^
AC(350)	68 ± 7	0.072 ± 0.007
AC(400)	74 ± 4	0.044 ± 0.002
AC(450)	83 ± 3	0.048 ± 0.002
AC(500)	80 ± 6	0.041 ± 0.003
AC(550)	88 ± 4	0.047 ± 0.002

^a^$\%{R}_{\mathrm{nicotine}}$ and $\%{R}_{\mathrm{nicotine},norm}$ are expressed as mean ± SD (n = 9). ^b^$\%{R}_{\mathrm{nicotine}}$ divided by *S*_BET_.

### Quantitative analysis of surface functional groups on ACs

Preliminarily, the functional group of ACs was investigated by FTIR (Figure S4). The broad peak at 3360 cm^−1^ can be assigned to O–H stretching of hydroxyl group. The small peak at 1700 cm^−1^ can be ascribed to C=O stretching in ketones, aldehydes, and carboxylic acids. The vibration at 1600 cm^−1^ can be assigned to aromatic C=C stretching. The peaks at 1160 and 970 cm^−1^ can be attributed to C–O stretching of a tertiary alcohol, and C=C bending in an alkene, respectively. However, the carbon materials have a strong absorption in the IR region^35^, the spectra of the functional groups of the obtained activated carbons have a low signal-to-noise ratio, which makes their determination difficult.

**Figure 2:**
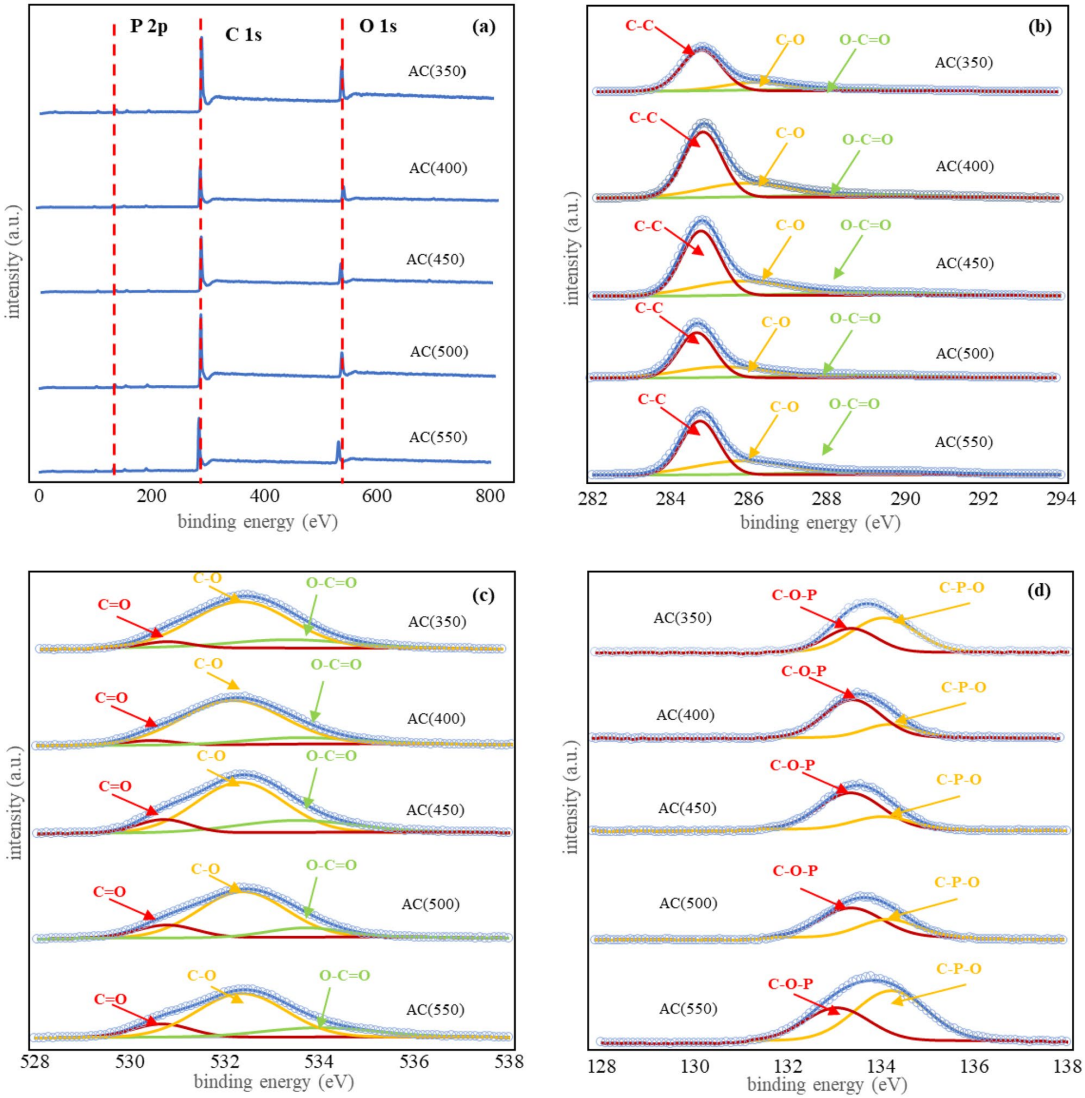
(**a**) XPS survey spectra, (**b**) C 1 s, (c) O 1 s, and (**d**) P 2p of the obtained ACs.

The chemical composition of the ACs was then investigated by X-ray photoelectron spectroscopy (XPS). The wide-scan spectra (Fig. 2a) show the presence of carbon (C 1s), oxygen (O 1 s), and phosphorus (P 2p). For the narrow scan, C 1 s peaks (Fig. 2b) at 284.8, 285.8, and 288.8 eV can be assigned to C–C, C–O, and O–C=O (COOH species), respectively. O 1s spectra (Fig. 2c) show the peaks at 530.3, 532.4 and 533.8 eV, assigned to C=O bonds (carbonyl C=O and carboxylic O=C–OH groups), C–O bonds (mainly hydroxyl C–OH and epoxy C–O–C), and O–C=O bonds (carboxylic and anhydride), respectively. P 2p spectra (Fig. 2d) can be deconvoluted to C-O-PO_3_ (133.0 eV) and C-PO_3_ (134.2 eV)^36,37^.

**Figure 3:**
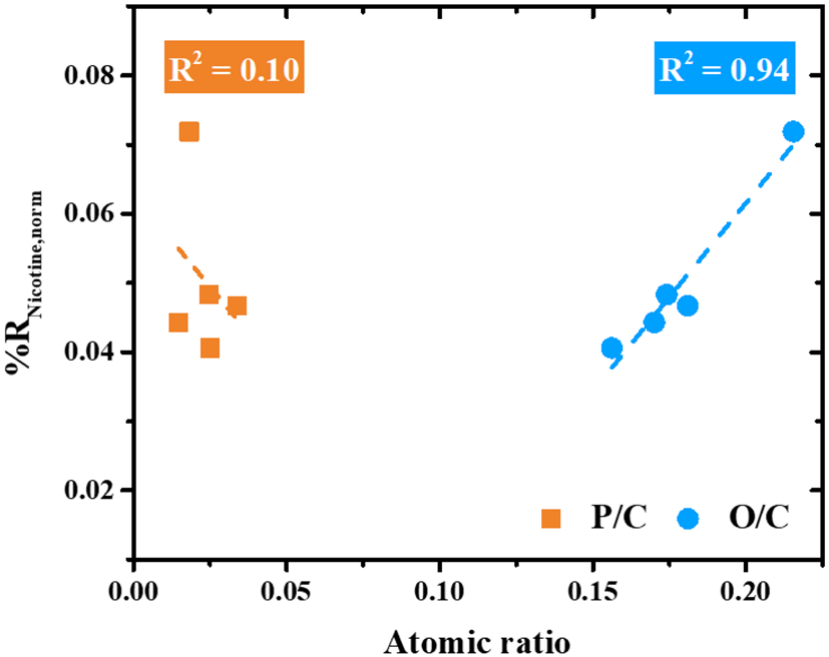
The plot of normalized nicotine reduction ($\%{R}_{\mathrm{nicotine},\mathrm{ norm}}$) versus ratio of O/C and P/C.

The atomic concentrations of each element (C, N, O, and P) are shown in Table S2, where the produced ACs are rich in carbon (> 80%). Oxygen is the second largest fraction at 13 – 17%. Nitrogen and phosphorus are of lesser importance compared to carbon and oxygen. Since the atomic concentration of nitrogen was relatively low (0.2 – 0.6%), the effects of nitrogen-containing functional groups were neglected in this study. As mentioned earlier, the adsorption capacity of nicotine depends on the surface functional groups, so oxygen and phosphorus would have an impact on nicotine reduction. To illustrate the relationship between nicotine removal and the surface functionality of each heteroatom, the normalized $\%{R}_{\mathrm{nicotine}}$ was plotted against the oxygen-carbon (O/C) and the phosphorus-carbon (P/C) contents (Fig. 3). It can be observed that the presence of oxygen has a strong influence on the adsorption of nicotine (R^2^ = 0.94). Phosphorus, on the other hand, shows an unclear effect on nicotine adsorption (R^2^ = 0.10) so that it was also neglected in this study. The oxygen-containing functional groups of ACs can be present in many forms, such as anhydride, phenol, quinone, carboxylic and lactone^28,38^. Unfortunately, XPS cannot fully assign a variety of complex oxygen functional groups because XPS is only a surface analysis. Therefore, TPD was employed to fully understand the surface chemistry of the obtained ACs.

**Figure 4:**
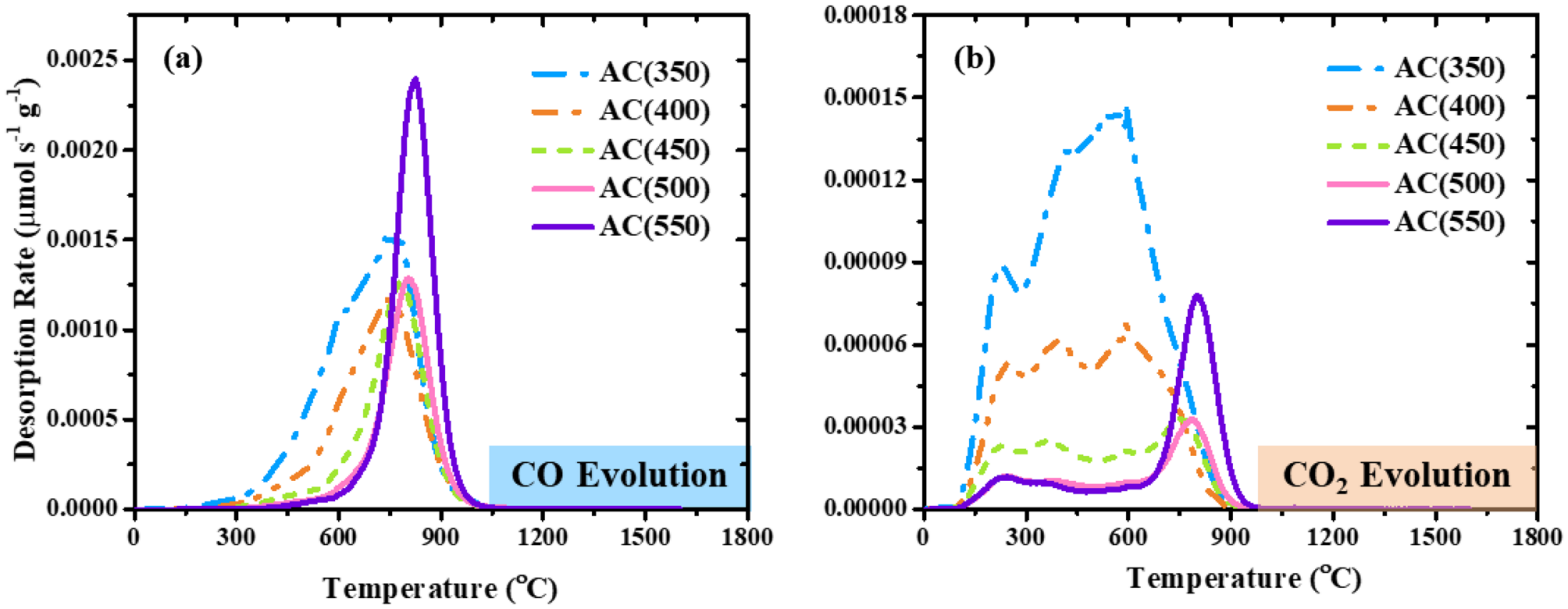
TPD spectra ((**a**) CO and (**b**) CO_2_) of the obtained ACs with different activation temperatures.

Figure 4 shows the TPD spectra of the obtained ACs with different activation temperatures. For the evolution of CO (Fig. 4a), the main contributions are the anhydride decomposition peak at 400 – 600 °C, the phenol/ether decomposition peak at 600 – 800 °C, and the quinone/carbonyl and phosphate groups decomposition peak above 800 °C^27,28,38–40^. The evolution of CO_2_ (Fig. 4b) results mainly from the decomposition of carboxylic acid at 100–400 °C, anhydride at 400–600 °C, and lactone at 600–800 °C^41,42^. The CO_2_ observed in the high temperature region above 800 °C is considered to originate from the desorption of phosphate groups^40^.

Both the CO and CO_2_ evolution spectra were deconvoluted to obtain qualitative and quantitative data on the oxygen-containing functional groups of the ACs. Table 3 shows the amounts of each functional group on the surface of the activated carbon obtained from the deconvolution spectra. The results show that the phenol, anhydride, and carboxyl content decreased with activation temperature, while the quinone/carbonyl (including phosphate groups) and lactone content was varied in each sample. The results indicate that phenol, anhydride, and carboxylic groups are decomposed at high activation temperatures due to their structural instability.

**Table 3:** The amounts of each functional group obtained from the deconvolutions of the TPD spectra.

Sample	Phenol/ether (mmol/g)	Quinone/carbonyl and phosphate (mmol/g)	Carboxylic (mmol/g)	Lactone (mmol/g)	Anhydride (mmol/g)
AC(350)	1.57	2.58	0.41	0.05	1.01
AC(400)	0.46	2.37	0.18	0.02	0.65
AC(450)	0.20	2.17	0.08	0.04	0.34
AC(500)	0.21	2.02	0.04	0.05	0.16
AC(550)	0.19	3.31	0.04	0.12	0.14

**Figure 5:**
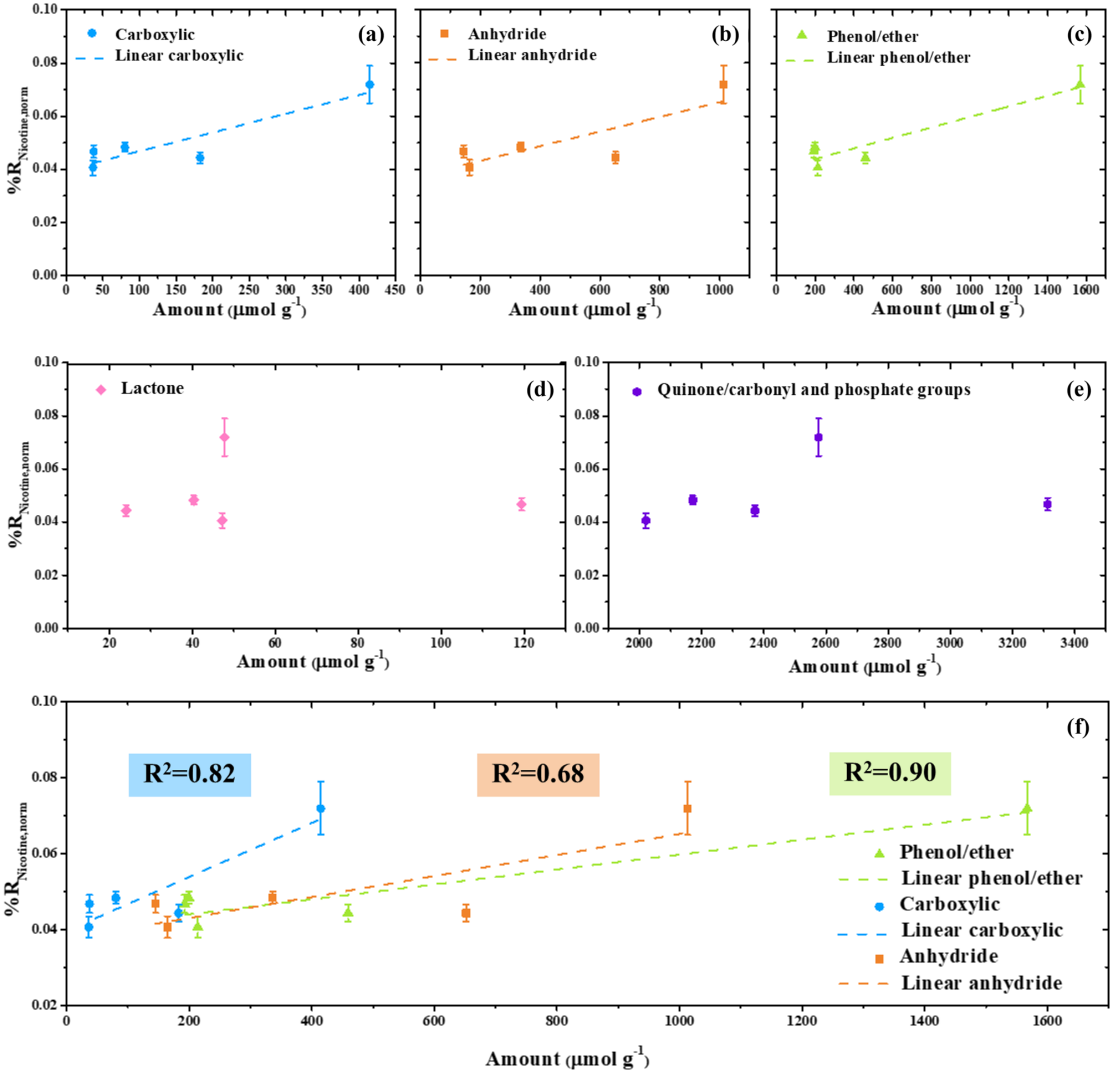
Plots of oxygen-containing surface functional groups. (**a**) Carboxylic acid, (**b**) anhydride, (**c**) phenol/ether, (**d**) lactone, (**e**) quinone/carbonyl and phosphate groups against the normalized nicotine reduction along with their linear regression fit, and (**f**) comparison on major functional groups that contribute to nicotine adsorption. Note that ($\%{R}_{\mathrm{nicotine},\mathrm{ norm}}$) is expressed as the mean ± SD (n = 9).

To further investigate the effects of oxygen-containing functional groups on the ability to remove nicotine, the amount of each functional group was plotted against the normalized nicotine reduction, as shown in Fig. 5. It can be seen that the normalized nicotine reduction increases with the amount of carboxylic (Fig. 5a), phenol/ether (Fig. 5b), and anhydride (Fig. 5c) groups. On the other hand, the effects of lactone (Fig. 5d) and carbonyl/quinone and phosphate groups (Fig. 5e) on nicotine removal are unclear, implying that neither functional group has a significant effect on nicotine adsorption. Therefore, only the effects of the carboxylic, phenol/ether, and anhydride groups were compared, as shown in Fig. 5f, with the carboxylic groups contributing the most to nicotine adsorption with the least amount required.

### Computational study on the influence of oxygen-containing functional groups on nicotine adsorption mechanisms

The hydrogen-terminated graphene sheet was used to represent the nanoscale surface structure of ACs, modified by substituting the selected oxygen-containing functional groups. It was found that the adsorption energy of the graphene and nicotine complexes was consistently negative, indicating the ability to adsorb nicotine on hydrogen-terminated graphene and oxygen-functionalized graphene, as listed in Table 4. The more negative the adsorption energy, the higher the adsorption efficiency. Consequently, the adsorption energy of carboxylic graphene is much lower than the others. This could be due to the high reactivity of the carboxylic group, which could enhance the interaction between nicotine and surface of ACs. Benzoquinone graphene, glutaric anhydride graphene, and δ-valerolactone graphene also exhibited considerably high nicotine adsorption efficiency with adsorption an energy of about − 10 kcal mol^−1^, which could be due to the reactivity of the carbonyl groups. On the other hand, hydroxylic graphene and hydrogen terminated graphene have similar nicotine adsorption efficiency with adsorption energy of about −8.3 kcal mol^−1^.

**Table 4:** Adsorption energies (Δ*E*) of nicotine on graphene with various oxygen surface functional groups.

Complex structures	Δ*E* (kcal mol^−1^)
Graphene/nicotine	− 8.32
Carboxylic graphene/nicotine	− 15.58
1,2-Benzoquinone graphene/nicotine	− 10.69
Glutaric Anhydride/nicotine	− 10.30
δ-Valerolactone graphene/nicotine	− 10.17
Hydroxylic graphene/nicotine	− 8.36

**Figure 6:**
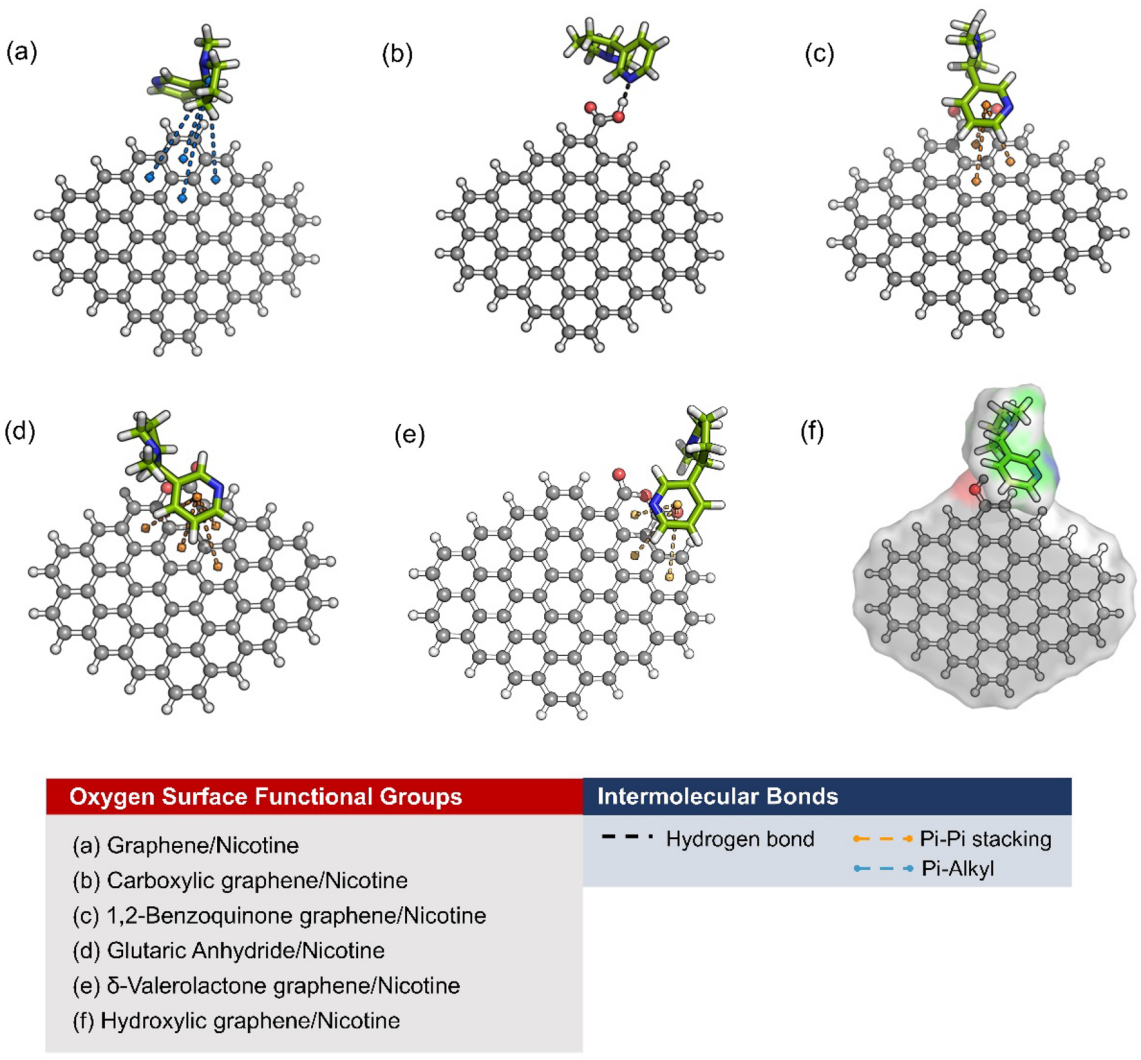
Intermolecular interaction between nicotine molecule and graphene with several types of surface functional groups: (**a**) pristine graphene, (**b**) carboxylic graphene, (**c**) 1,2-benzoquinone graphene, (**d**) glutaric anhydride, (**e**) δ-valerolactone, and (**f**) hydroxylic graphene. Hydrogen-terminated graphene and oxygen-functionalized graphene were presented as ball and stick models, while the nicotine molecule is presented as a stick model. The black, orange, and blue dash lines were used to represent hydrogen bond, π−π stacking interaction, and π-alkyl interaction, respectively. Transparent surfaces were used to show the van der Waals surfaces between nicotine molecule and graphene.

To further investigate the adsorption ability of nicotine on the oxygen-containing functional groups of the graphene structure, the intermolecular interactions between the nicotine molecule and each graphene surface were observed (Fig. 6). As expected, hydrogen bonds exist between the hydrogen atom of the carboxylic group of graphene and the pyridinic nitrogen atom of the nicotine molecule. The strong hydrogen bonding with an N–H distance of 1.64 Å facilitated the adsorption of nicotine on carboxylic graphene (Fig. 6b). Interestingly, the reactivity of the carbonyl groups of benzoquinone and δ-valerolactone did not play a significant role in nicotine adsorption, as only strong π-π-stacking interactions (3.4–4.9 Å) were formed between the pyridine ring of nicotine and the benzene, benzoquinone, and δ-valerolactone rings on graphene (Fig. 6c,e). Similar behavior can be observed for the glutaric acid-graphene/nicotine complex (Fig. 6d). For the hydrogen-terminated graphene/nicotine complex (Fig. 6a), the weak π-alkyl interactions (4.3–5.0 Å) between the pyrrole ring of nicotine and the benzene rings on graphene were found instead. Conversely, hydroxylic graphene had no prominent interaction with the nicotine molecule. Although, the pyrrolic and pyridinic nitrogen atoms of the nicotine molecule are rich in positive charges and normally act as electron acceptors, the nicotine molecule could be attracted to the hydroxyl graphene via a weak van der Waals interaction, the van der Waals surface with a probe radius of 1.4 Å, as shown in Fig. 6f.

**Figure 7:**
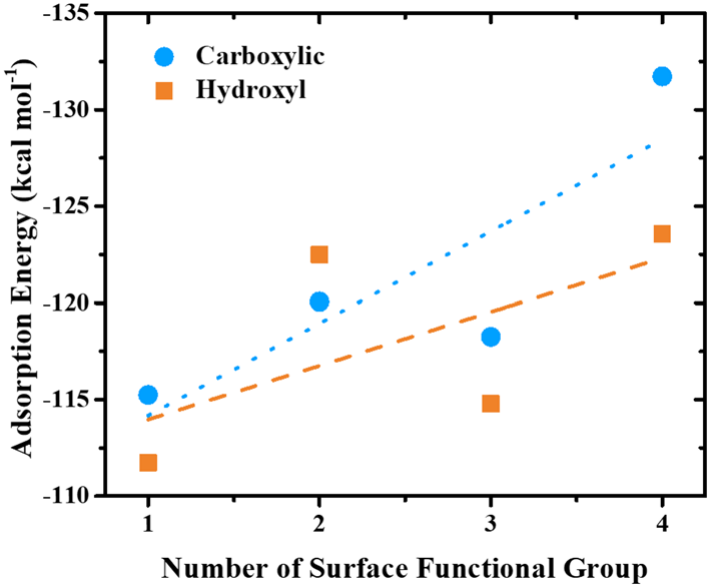
Effect of nicotine adsorption based on the amount of selected oxygen surface functional groups.

Although the oxygen-containing functional groups could facilitate the adsorption of nicotine, the 1:1 adsorption energy previously reported cannot represent the effect of the amount of oxygen functional groups on nicotine adsorption. Therefore, the additional semi-empirical calculation was performed using the PM7 model in the Gaussian 16 program for the selected oxygen-containing functional groups with the highest and lowest adsorption capacities, the carboxylic group, and the hydroxyl groups, respectively. Simulations were performed by varying the amount of each oxygen-containing functional group from 1 to 4 and keeping the number of nicotine molecules constant at 5. Geometry optimization for each complex system was then investigated. As a result, the adsorption energies between 5 nicotine molecules were plotted with the variation of the selected oxygen-containing functional groups in Fig. 7. The linear regression trend lines were also displayed to facilitate observation of the trend in nicotine adsorption. It can be seen that as the number of surface functional groups increases, the carboxylic group has a higher tendency to adsorb nicotine than the hydroxyl group. The calculation suggests that other oxygen complexes have higher adsorption capacity with an increasing number of functional groups. However, their adsorption efficiencies are still lower than that of the carboxylic group. This is in good agreement with the results of the TPD analyses, where the carboxylic group shows a superior effect on nicotine adsorption. It has been reported that phenolic groups could increase the adsorption capacity of nicotine in the liquid phase, while carboxylic groups could hinder adsorption as they could combine with water molecules through hydrogen bonds and block the pores^12^. However, the adsorption of nicotine in cigarette smoke with low water content in this study could eliminate this drawback, and further highlight the effects of carboxylic groups on the enhancement of nicotine adsorption.

## Conclusions

In this study, activated carbons (ACs) with variable surface functional groups and porosity were successfully prepared from dried hemp stem by chemical activation with phosphoric acid. The removal of nicotine from cigarette mainstream smoke was 88% with the AC at an activation temperature of 550 °C, although its specific surface area was lower than that activated at 500 °C. This indicates that the adsorption mechanism of nicotine was not only physical adsorption but also chemical adsorption, which was influenced by the surface chemistry of the ACs. According to XPS and TPD-MS analyses, the oxygen-containing functional groups showed a high contribution to nicotine adsorption. We have found that carboxylic groups possess the superior ability to adsorb nicotine among the others. In addition, computational analysis of the effects of oxygen-containing surface functional groups further supports that the carboxylic groups provide the lowest adsorption energy, implying the highest ability to adsorb nicotine over other functional groups. Therefore, we suggest that not only a high specific surface area of ACs, but also an oxygen-containing surface functional group, especially the carboxyl group, could facilitate the adsorption ability of nicotine.

### Supplementary Information

Supplementary Information.

## Data Availability

The datasets generated during and/or analysed during the current study are available from the corresponding author on reasonable request.
