# Supplementary material for: Insight into the effects of different oxygen heteroatoms on nicotine adsorption from cigarette mainstream smoke
Source: Sci Rep. 2023 Sep 15;13:15311. doi: 10.1038/s41598-023-42188-w (PMC10504273; doi:10.1038/s41598-023-42188-w)
Supplement: Supplementary file 1 — Supplementary Information. [file 41598_2023_42188_MOESM1_ESM.docx]

Supporting Information

**Insight into the effects of different oxygen heteroatoms on nicotine adsorption from cigarette mainstream smoke**

Phongphot Sakulaue^a^, Kulpavee Jitapunkul^a^, Parinya Inthasuwan^b^, Hiromu Takano^c^, Takafumi Ishii^c^, Kanokwan Kongpatpanich^d^, Kajornsak Faungnawakij^e^, Metta Chareonpanich^f^, Khanin Nueangnoraj*^a^

*^a^ School of Bio-Chemical Engineering and Technology, Sirindhorn International Institute of Technology, Thammasat University, Pathum Thani, 12120, Thailand*

*^b^ Sustainable Energy and Resources Engineering, Faculty of Engineering, Kasetsart University, Bangkok, 10900, Thailand*

*^c^ International Research and Education Center for Element Science, Faculty of Science and Technology, Gunma University, 1–5–1 Tenjincho, Kiryu, Gunma, 376–8515, Japan*

*^d^ Department of Materials Science and Engineering, School of Molecular Science and Engineering, Vidyasirimedhi Institute of Science and Technology, Rayong, 21210, Thailand*

*^e^ National Nanotechnology Center, National Science and Technology Development Agency, Pathum Thani 12120, Thailand*

*^f^ Department of Chemical Engineering, Faculty of Engineering, Kasetsart University, Bangkok, 10900, Thailand*

***Corresponding author:** K. Nueangnoraj (khanin@siit.tu.ac.th)


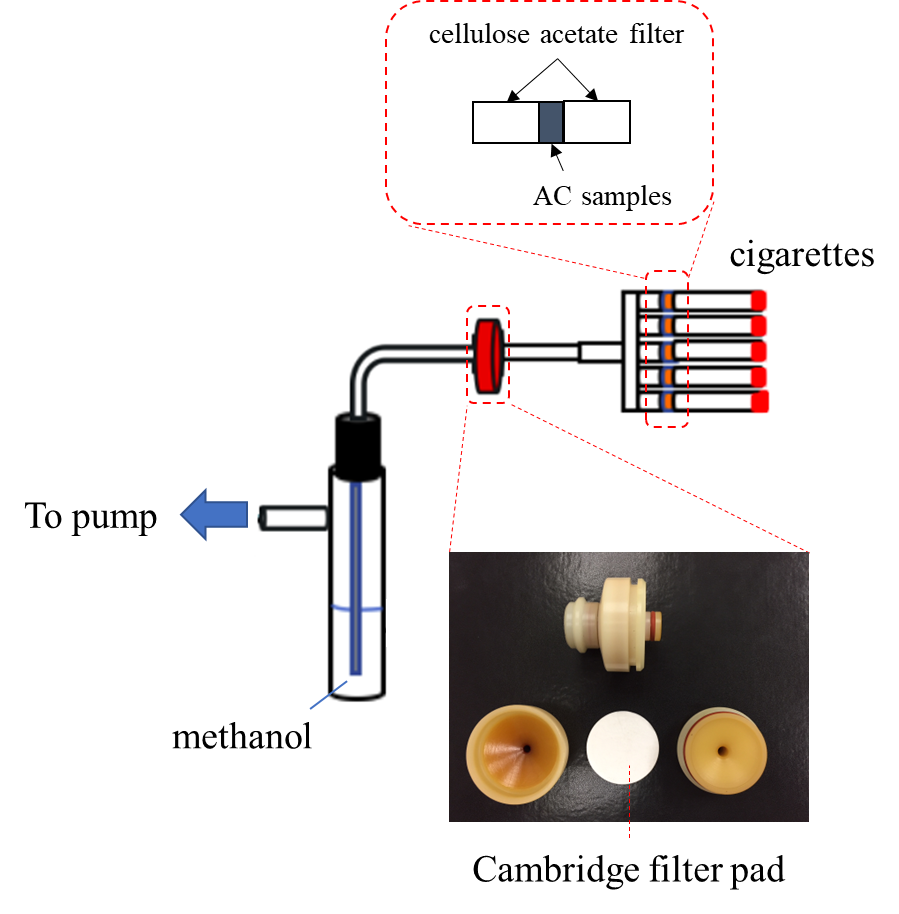


**Figure S1**. A schematic diagram of the laboratory-scale smoking apparatus.

**Analysis of nicotine**

The amount of nicotine that was trapped in methanol solution was analyzed by gas chromatography – flame ionization detector (GC-FID). The column was RTX-1 fused with silica gas chromatography capillary column (Restek, 30 m x 0.32 mm ID with the thickness of 0.5 µm) coated with 100% dimethyl polysiloxane. Helium was used as a carrier gas at a linear velocity of 2 ml min^-1^. Air and hydrogen flow rates were 450 and 45 ml min^-1^, respectively. The split ratio was 20:1. The injector was held isothermally at 250 °C. The column temperature program was set as follows: initial temperature 160 °C held for 3 min, then increased at 15 °C min^-1^ up to 230 °C. The FID was maintained at 300 °C. All results were performed in triplicate. Calibration curves were constructed in a range of 0.2 – 1.0 mg ml^-1^ from a standard nicotine solution (**Figure S2**).


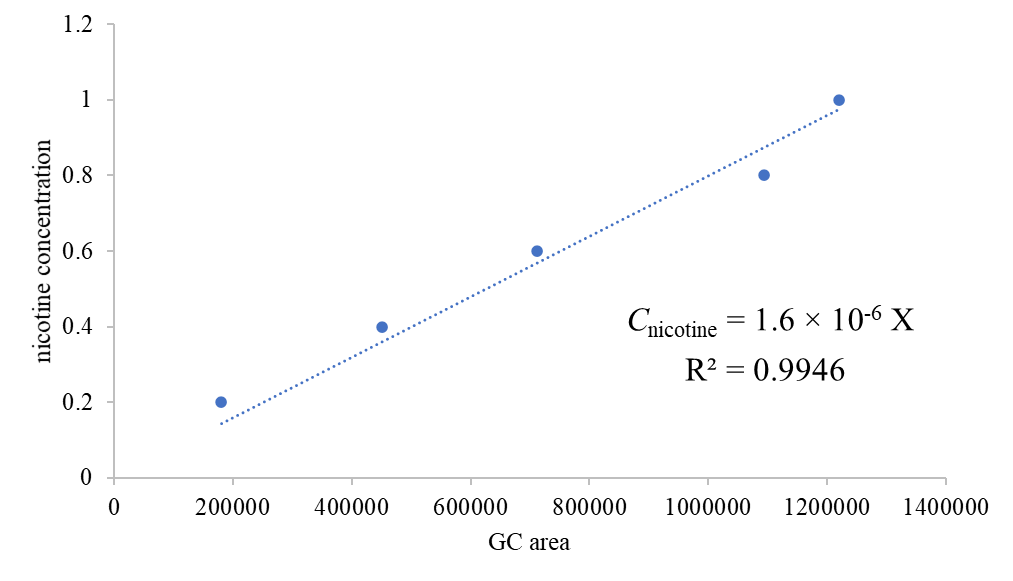


**Figure S2.** Calibration curve of nicotine in methanol solution.

**Table S1**. Time, area, concentration of nicotine, and amount of nicotine adsorbed from gas chromatography.

| **sample** | **average time**  **(min)** | **average area**  **(μV s^-1^)** | $\boldsymbol{C}_{\mathbf{nicotine,}}$  **(mg cigarette^-1^)** | **amount of nicotine adsorbed**  **(mg cigarette^-1^)** |
| --- | --- | --- | --- | --- |
| SMS (red) | 4.32 | 1044564 | 1.67 | – |
| AC(350) | 4.32 | 334685 | 0.53 | 1.14 |
| AC(400) | 4.32 | 275749 | 0.44 | 1.23 |
| AC(450) | 4.32 | 176411 | 0.28 | 1.39 |
| AC(500) | 4.32 | 212709 | 0.34 | 1.33 |
| AC(550) | 4.32 | 128025 | 0.20 | 1.47 |


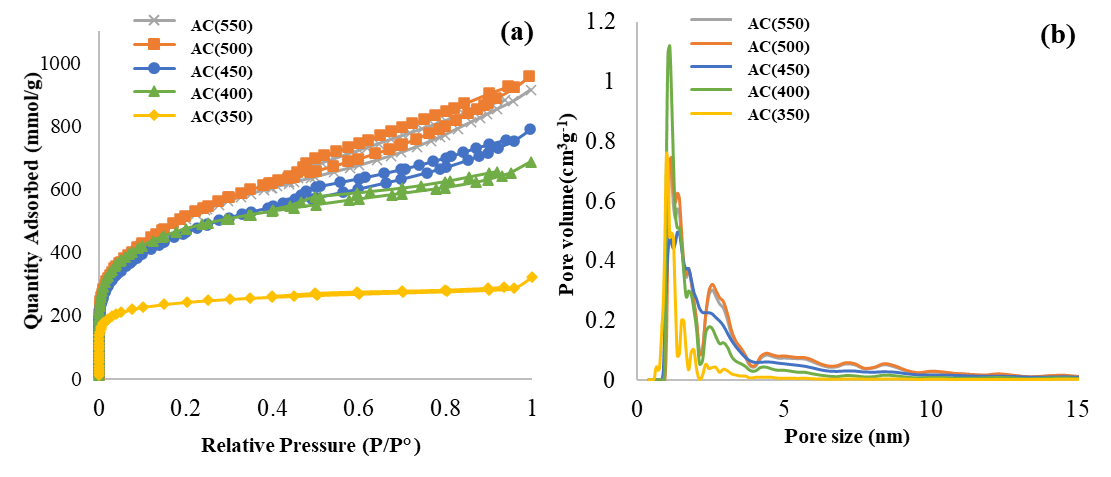


**Figure S3***.* (a) N_2_-sorption isotherms and (b) DFT pore size distributions of the obtained ACs.


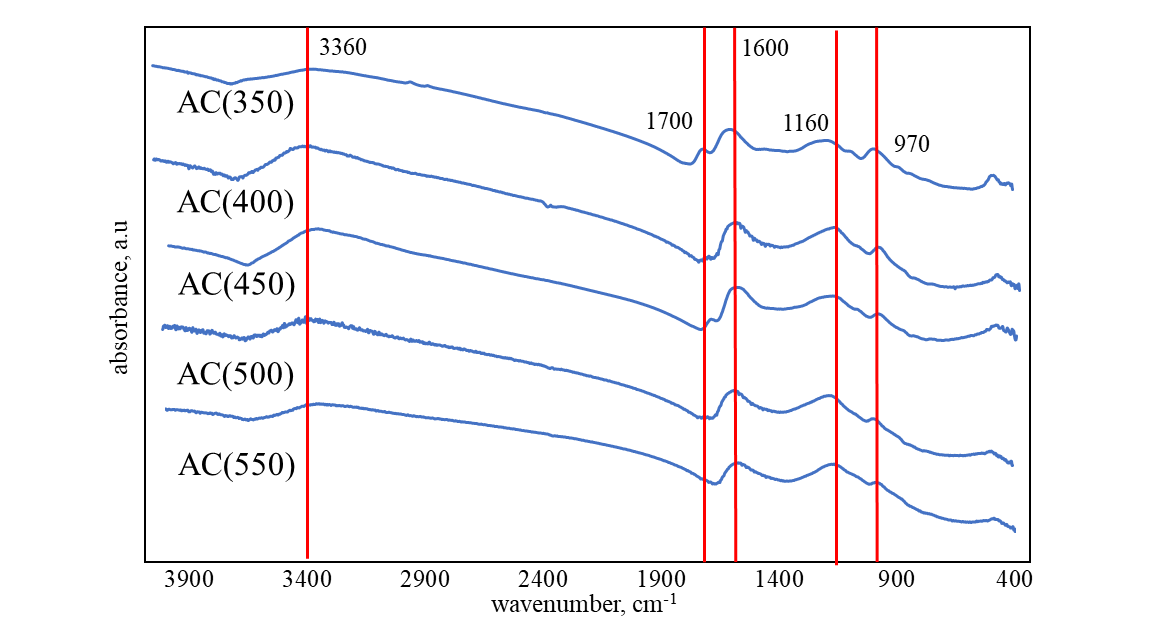


**Figure S4**. FTIR spectra of the obtained ACs.

**Table S2**. The atomic concentration of each element analyzed by XPS.

| sample | %at | | | | O/C | P/C |
| --- | --- | --- | --- | --- | --- | --- |
|  | C | O | N | P |  |  |
| AC(350) | 80.7 | 17.4 | 0.40 | 1.47 | 0.215 | 0.0182 |
| AC(400) | 84.1 | 14.3 | 0.33 | 1.24 | 0.170 | 0.0147 |
| AC(450) | 82.9 | 14.4 | 0.58 | 2.05 | 0.174 | 0.0247 |
| AC(500) | 84.4 | 13.2 | 0.24 | 2.12 | 0.156 | 0.0251 |
| AC(550) | 82.0 | 14.8 | 0.37 | 2.79 | 0.180 | 0.0340 |

**Table S3.** The amount of each functional group obtained from the peak deconvolution of CO and the CO_2_ evolution.

| **sample** | **CO**  **(mmol/g)** | **anhydride**  **(mmol/g)** | **phenol**  **(mmol/g)** | **quinone**  **(mmol/g)** | **CO_2_**  **(mmol/g)** | **carboxylic**  **(mmol/g)** | **anhydride**  **(mmol/g)** | **lactone**  **(mmol/g)** |
| --- | --- | --- | --- | --- | --- | --- | --- | --- |
| AC(350) | 4.82 | 0.68 | 1.57 | 2.58 | 0.80 | 0.42 | 0.34 | 0.05 |
| AC(400) | 3.29 | 0.46 | 0.46 | 2.37 | 0.40 | 0.18 | 0.19 | 0.02 |
| AC(450) | 2.64 | 0.27 | 0.20 | 2.17 | 0.19 | 0.08 | 0.07 | 0.04 |
| AC(500) | 2.36 | 0.13 | 0.21 | 2.02 | 0.12 | 0.04 | 0.04 | 0.05 |
| AC(550) | 3.62 | 0.12 | 0.19 | 3.31 | 0.18 | 0.04 | 0.03 | 0.12 |


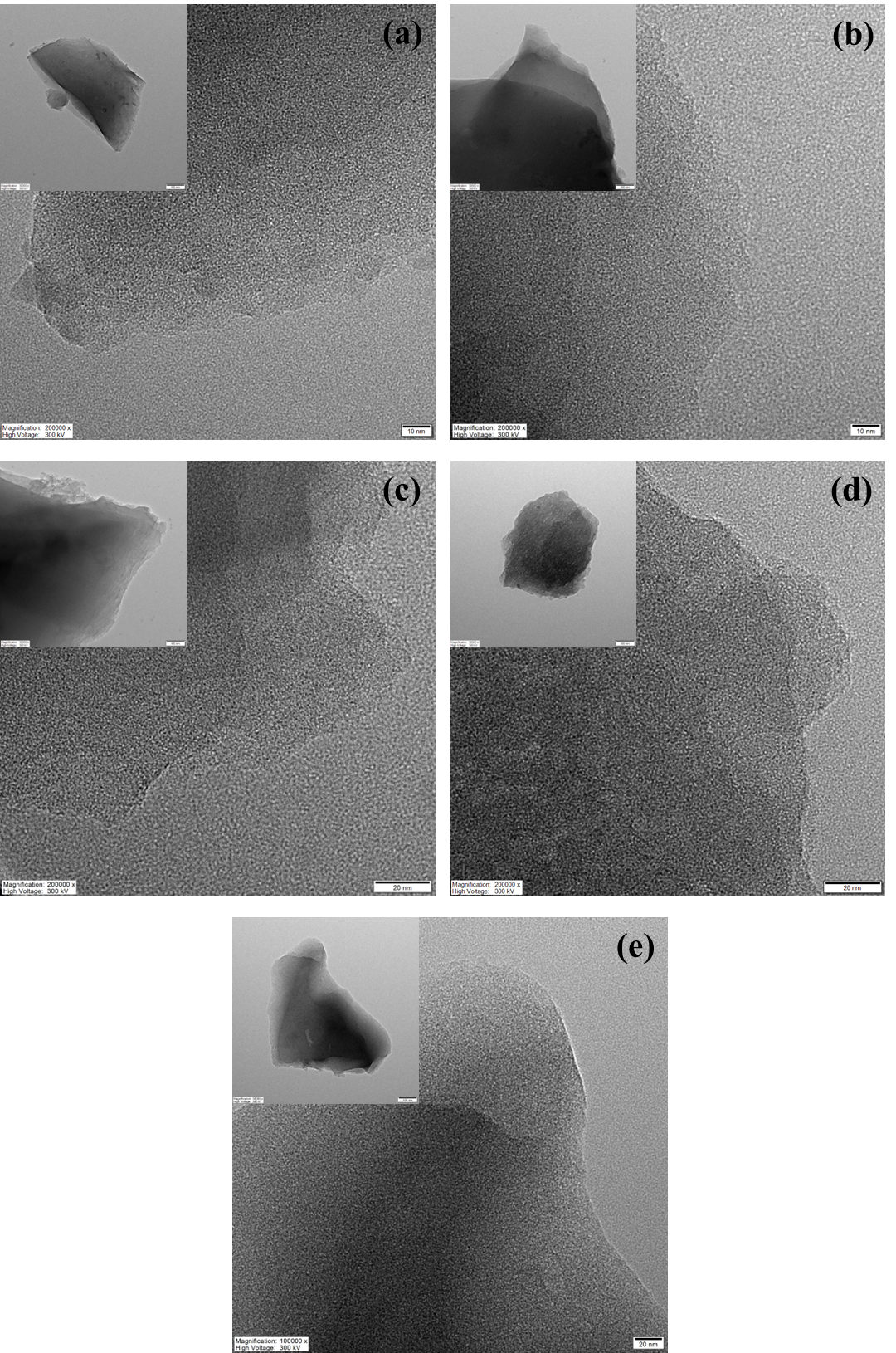


**Figure S5**. High resolution TEM images of the obtained ACs: (a) AC(350), (b) AC(400), (c) AC(450), (d) AC(500), and (e) AC(550). The inserts are those with low resolution.
